# Clinical laboratory reference values in adults in Kisumu County, Western Kenya; hematology, chemistry and CD4

**DOI:** 10.1371/journal.pone.0249259

**Published:** 2021-03-30

**Authors:** Valentine Sing’oei, Jew Ochola, John Owuoth, June Otieno, Eric Rono, Ben Andagalu, Lucas Otieno, Chiaka Nwoga, Nathanial K. Copeland, John Lawlor, Adam Yates, Michelle Imbach, Trevor A. Crowell, Leigh Anne Eller, Edwin Kamau, Kayvon Modjarrad, Jessica Cowden, Julie Ake, Merlin L. Robb, Christina S. Polyak

**Affiliations:** 1 HJF Medical Research International, Kisumu, Kenya; 2 U.S. Army Medical Research Directorate–Africa, Kisumu, Kenya; 3 Kenya Medical Research Institute, Kisumu, Kenya; 4 U.S. Military HIV Research Program, Walter Reed Army Institute of Research, Silver Spring, MD, United States of America; 5 Henry M. Jackson Foundation for the Advancement of Military Medicine, Bethesda, MD, United States of America; 6 Emerging Infectious Diseases Branch, Walter Reed Army Institute of Research, Silver Spring, MD, United States of America; Oregon State University, UNITED STATES

## Abstract

**Background:**

Clinical laboratory reference intervals (RIs) are essential for diagnosing and managing patients in routine clinical care as well as establishing eligibility criteria and defining adverse events in clinical trials, but may vary by age, gender, genetics, nutrition and geographic location. It is, therefore, critical to establish region-specific reference values in order to inform clinical decision-making.

**Methods:**

We analyzed data from a prospective observational HIV incidence cohort study in Kombewa, Kenya. Study participants were healthy males and females, aged 18–35 years, without HIV. Median and 95% reference values (2.5^th^ percentile to 97.5^th^ percentile) were calculated for laboratory parameters including hematology, chemistry studies, and CD4 T cell count. Standard Deviation Ratios (SDR) and Bias Ratios (BR) are presented as measures of effect magnitude. Findings were compared with those from the United States and other Kenyan studies.

**Results:**

A total of 299 participants were analyzed with a median age of 24 years (interquartile range: 21–28). Ratio of males to females was 0.9:1. Hemoglobin range (2.5^th^—97.5^th^ percentiles) was 12.0–17.9 g/dL and 9.5–15.3 g/dL in men and women respectively. In the cohort, MCV range was 59-95fL, WBC 3.7–9.2×10^3^/μL, and platelet 154–401×10^3^/μL. Chemistry values were higher in males; the creatinine RI was 59–103 μmol/L in males vs. 46–76 μmol/L in females (BRUL>.3); and the alanine transferase range was 8.8–45.3 U/L in males vs. 7.5–36.8 U/L in females (SDR>.3). The overall CD4 T cell count RI was 491–1381 cells/μL. Some parameters including hemoglobin, neutrophil, creatinine and ALT varied with that from prior studies in Kenya and the US.

**Conclusion:**

This study not only provides clinical reference intervals for a population in Kisumu County but also highlights the variations in comparable settings, accentuating the requirement for region-specific reference values to improve patient care, scientific validity, and quality of clinical trials in Africa.

## Introduction

The high burden of preventable infectious diseases in resource-constrained countries, particularly in sub-Saharan Africa (SSA), and the recent rise in non-communicable diseases [1] has led to an increase in research studies and clinical trials conducted in these regions [2–4]. Clinical laboratory RIs are necessary for screening potential study participants, assessing adverse events, monitoring response to therapy in clinical trials and routine patient care. However, the most commonly used references are derived from European and United States (US) populations [5]. Due to region-specific variation in RIs, reliance on standards established in Europe and the US could jeopardize study validity, increase the risk of negative outcomes, and cause social harm if potential participants are mislabeled as “unhealthy” and ineligible for study participation [6–8]. Consequently, a number of African countries involved in clinical trials have conducted research to establish region-specific RIs derived from SSA populations [7–14]. Even within SSA, regional variation would be expected; thus, it is useful to develop locally-derived standards for a potential study population of a given region.

One study in western Kenya of reference intervals for clinical laboratory parameters showed that more than 58% of participants in a clinical trial would have been deemed ineligible during screening if US-derived reference values were used [13]. A phase I HIV vaccine trial conducted in Nairobi reported that 61% of screening failures were due to laboratory abnormalities [15]. Laboratory values that fall outside of established reference values based on resource-rich settings, but are typical of healthy individuals in the populations being studied, can lead to a requirement for larger numbers of volunteer screenings and longer recruiting periods, resulting in increased research costs within already resource-constrained settings. Additionally, utilizing reference intervals derived from populations other than the study population may result in the erroneous grading of adverse events (AEs) due to population-specific variations in responses to drugs and vaccines [16, 17]. Furthermore, in general patient care, inappropriately applied external reference values may result in inappropriate and/or costly management decisions, such as the addition of unnecessary follow-on testing or the inappropriate dosing of medications. Therefore, to mitigate such outcomes, it is necessary to establish region-specific clinical laboratory RIs and toxicity tables.

Kombewa, Kenya is a location for a variety of observational and interventional studies investigating diseases that are endemic to sSA, such as infectious diseases including malaria, HIV and tuberculosis. However, hematologic, clinical chemistry, and CD4 reference intervals for adults at the Kombewa Clinical Research Center (KCRC) have not been systematically studied and established. Depending on the study protocol, the values that are currently used in the area are adopted from the Harriet Lane handbook [18] and Beckman Coulter [19], both of which are derived from studies of Caucasian populations in resource-rich settings.

Locally-derived reference intervals for the study population could facilitate shorter clinical trial recruitment timelines, increase accuracy in monitoring for and evaluation of adverse events, and improve routine general patient management. The aim of this study was to establish clinical laboratory reference intervals for hematology, clinical chemistry, and CD4 absolute values in healthy females and males aged 18 to 35 years in Kisumu County, Kenya.

## Methods

### Study area, population and participant recruitment

**Fig 1:**
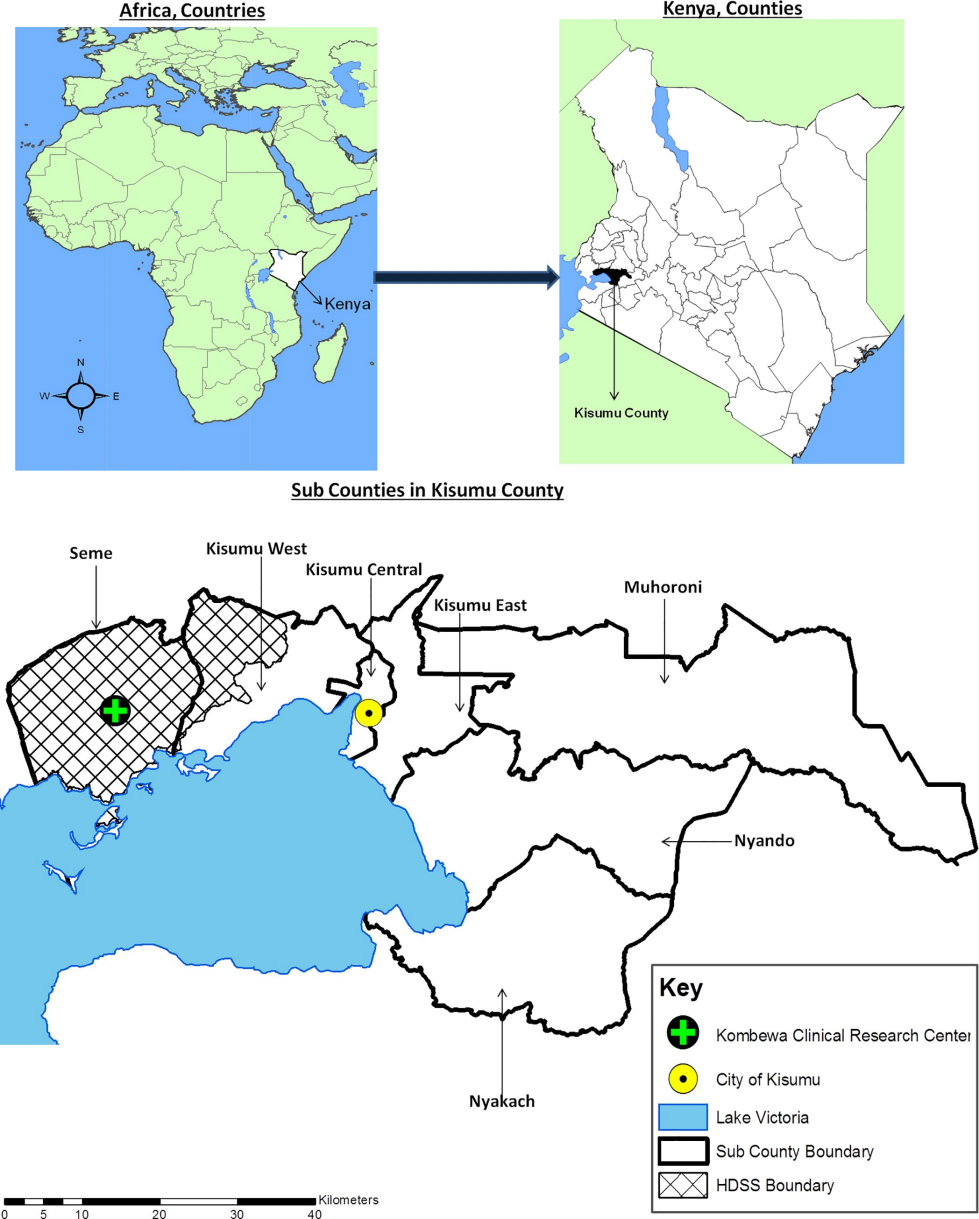
Geographic location of the study site. The map shows the context within Africa (top left) and Kenya (top right), and detailing Kisumu County including the Kombewa Clinical Research Center and the Kombewa Health and Demographics Surveillance System (HDSS) (bottom). The map was custom generated in-house using the ArcGIS Version 10.3.1 (Environmental Systems Research Institute, Red-lands, CA, USA) software.

These analyses utilized data from participants enrolled from February 2017 through May 2018 into a prospective cohort study to determine HIV incidence and assess the site’s suitability for future HIV-prevention trials in Kisumu County, Kenya. The city of Kisumu is the capital of Kisumu County, is located at the coordinates 0°6′S 34°45′E at an altitude of 1,131 m (3,711 ft.), and has a population of about 1.1 million [20]. Kisumu County is a malaria-endemic region [21] with a high HIV prevalence of 16.3% which is about three times the national prevalence of 4.9% [22]. Within the western portion of the county, the KCRC has a Health and Demographics Surveillance System (HDSS) that covers an area of about 369 km^2^ with a population of 154,140 as of December 2019. The study population included in these analyses were recruited from the Kombewa HDSS (30%) and other sub-counties (70%) within Kisumu County (Fig 1).

One of the secondary objectives of the cohort study was to establish local RIs for hematology, chemistry, and CD4 T cell counts. All participants underwent medical history-taking, physical examination and tests for HIV, malaria, syphilis, schistosomiasis, and hepatitis B and C. Female participants also underwent pregnancy testing. Participants who were living with HIV, pregnant, or had significant medical conditions were excluded from the study. Additionally, participants with syphilis, hepatitis B, hepatitis C, or malaria, and/or who were missing key laboratory result were excluded from the analyses to establish reference laboratory values for a healthy population. Samples for hematology, chemistry, and CD4 were collected solely at enrollment visit until sufficient data were available for RI calculation [23].

### Sample collection procedures for HIV testing and other laboratory testing

HIV testing was carried out in accordance with the Kenyan Ministry of Health serial HIV testing algorithm [24] with finger prick collection of whole blood for rapid testing using the Determine™ assay (Abbott Laboratories, Matsudo, Japan), with a reactive result confirmed using First Response® (Premier Medical Corporation limited, Mumbai, India). Discordant HIV results were further tested using fourth-generation Geenius™ HIV 1/2 Confirmatory Assay (Bio-Rad Laboratories, Johannesburg, South Africa). Venipuncture was performed for collection of whole blood and serum using Vacutainer® blood collection tubes (Beckton Dickinson, Plymouth, United Kingdom). Hematology parameters such as absolute WBC counts and percentages for WBC differentials (neutrophils, lymphocytes, monocytes, eosinophils, and basophils), red blood cells (RBC) with related parameters (hemoglobin, hematocrit, mean corpuscular volume [MCV], mean corpuscular hemoglobin [MCH], mean corpuscular hemoglobin concentration [MCHC], and red cell distribution width [RDW]), and platelet counts were determined from whole blood using a Coulter Ac•T™ 5diff CP analyzer (Beckman Coulter, Paris, France). The tests were performed within 24 hours of sample collection as instructed by the manufacturer. Samples for clinical chemistry (alanine aminotransferase [ALT] and creatinine) were allowed to clot for a minimum of 30 minutes, centrifuged, and the serum analyzed within the same day of separation. ALT and creatinine tests were analyzed using the cobas® c 311 biochemistry analyzer (Roche, Mannheim, Germany) according to the manufacturer’s instructions. Absolute CD4 T cell counts were determined using a point of care Pima™ Analyzer (Alere Technologies, Jena, Germany) within 36 hours of sample collection following manufacturers recommendations.

Pregnancy testing in female participants was performed using the QuickVue® rapid diagnostic test (Quidel Corporation, San Diego, USA) on urine samples in accordance with manufacturer’s recommendations. Malaria testing was performed by expert blood film light microscopy. The malaria result was confirmed negative after reading at least 200 high-power fields by two independent operators. Discrepant results were resolved by a third reader [25]. Syphilis was evaluated using Impact™ RPR carbon agglutination test kit (Alere Technologies, Jena, Germany) in accordance with manufacturer’s recommendations. Hepatitis B Surface Antigen (HBsAg) was tested using HBsAg 3.0 ELISA kit (Bio-Rad, Johannesburg, South Africa) and hepatitis C was tested using Ortho® HCV3.0 ELISA (Ortho-Clinical Diagnostics Inc, Raritan, NJ) in accordance with manufacturer’s recommendations.

### Quality control

For all laboratory tests performed, including evaluation for potential infectious diseases, quality control was performed. This included testing known positive and negative samples for qualitative tests and samples with known concentrations/values for quantitative tests. Test results were considered invalid if the quality control results failed. Additionally, the laboratory that performed all testing is enrolled in external quality assurance testing programs including the United Kingdom National External Quality Assurance Service; Royal College of Pathology, Australia; and Oneworld Accuracy, Canada. The laboratory complies with the principles of Good Clinical Laboratory Practices (GCLP). All analyzers used were validated appropriately in accordance with GCLP requirements.

### Statistical analyses

Data were transformed via the Box-Cox [26] method in SAS 9.4—TRANSREG Procedure. Unusual values (“outliers”), defined by Tukey as measurements beyond the 75^th^/25^th^ percentile ± 1.5 x interquartile range (IQR), were identified for elimination [27] after Box Cox Transformation. Data were analyzed to establish the middle 95%: 2.5^th^ percentile to 97.5^th^ percentile of those remaining after using Tukey’s elimination point [23]. Female and male values were compared using the two-tailed Wilcoxon rank sum test.

In order to depict the practical magnitude of the statistical difference between male and female RIs, we calculate the standard deviation ratio (SDR) using a 1-way ANOVA approach to determine the within-group deviation from the overall median value [28–30]. We use the critical value of SDR>0.3 to signify significant difference between sex-specific assay medians to warrant sex-specific RIs. As SDR is a measurement of different from the point of centrality, we also include a comparison metric, “Bias Ratio” (BR), which may more appropriately characterize difference between groups at the extremes of the distribution [30]. The BR calculation examines the dispersion for the RI at the extremes for male and female values compared to the extremes for the combined population divided by standard deviation (SD) comprising reference interval (i.e. UL = mean+1.96SD, UL = mean−1.96SD):

$$BRul=\frac{ULm-ULf}{(ULmf-LLmf)/3.92}$$

A BR value of |BR|>0.375 for upper or lower values indicates the need for partitioning by group.

### Ethical considerations

Ethical approval for the study was obtained from KEMRI Scientific and Ethics Review Unit, Nairobi, Kenya, and Walter Reed Army Institute of Research Institutional Review Board, Silver Spring, MD, USA. Written informed consent was obtained from each participant prior to enrollment.

## Results

Of the 579 HIV uninfected participants enrolled into the HIV incidence cohort study, 299 participants were included in the analyses of which 142 (47.5%) were males and 157 (52.5%) were females. The median age was 24 years (IQR, 21–28).

Of the 280 participants excluded from the analyses, 83 (29.6%) were excluded due to infection with malaria, syphilis, hepatitis B, or hepatitis C; 165 (58.9%) were excluded due to unavailability of key laboratory results; and 32 (11.4%) had both infection and unavailable results. The sample size was less than 299 for some of the lab tests since the interquartile range of 75%-25% removed outliers.

Table 1 summarizes the reference values for the hematology and absolute CD4 count parameters of the eligible participants in the cohort. There were significant gender differences in hemoglobin, hematocrit and RBC; Males had higher RIs compared to females (*p*<0.0001; SDR>1.0). Significant differences were also observed for WBCs, lymphocytes,(p<0.05, SDR>0.3) and platelets (p<0.05,BRUL0.375) where females had higher RIs; Neutrophil levels were different between men and women according to the Wilcoxon test, but the SDR and BR tests indicate the differences are not likely to be of practical magnitude. Absolute eosinophils were higher in males (*p* = 0.0077; BRUL>2.0). Table 2 shows the ALT and creatinine median and RIs where both parameters were significantly different between genders with higher values in males (p<0.0001, SDR>0.3). In males, the upper limit for ALT and creatinine were 1.2-fold and 1.35-fold higher respectively.

**Table 1:** Hematological reference intervals and CD4 absolute count (median and 2.5^th^—97.5^th^ percentiles) derived from healthy young adults in Kisumu County.

Parameter	Total		Male		Female		*P*-values**	SDRsex	BRLL	BRUL
	n	Median (range*)	n	Median (range*)	n	Median (range*)				
Hemoglobin (g/dL)	298	14.3 (10.6–17.5)	142	15.7 (12.0–17.9)	156	13.4 (9.5–15.3)	**< .0001**	**1.16**	**1.42**	**1.48**
Hematocrit (%)	296	43.6 (35.3–53.4)	141	48.1 (38.5–54)	155	41.1 (32.7–47.3)	**< .0001**	**1.39**	**1.26**	**1.45**
RBC (10^6^/μL)	294	5.36 (4.35–6.61)	138	5.67 (4.8–6.73)	156	4.94 (4.19–6.29)	**< .0001**	**1.06**	**1.06**	**0.76**
MCH (pg)	290	27.1 (20.7–31.6)	140	27.7 (21.7–31.6)	150	26.9 (20–32)	**0.0357**	0.22	**0.61**	-0.14
MCV (fL)	296	83 (59–95)	140	83 (66–95)	156	82 (56–98)	0.1694	0.12	**1.09**	-0.33
MCHC (g/dL)	292	32.7 (29.9–34)	139	32.8 (29.9–34.2)	153	32.6 (29.7–34)	**0.0086**	0.17	0.19	0.19
RDW_CV (%)	292	11.9 (10–15.4)	140	12 (9.8–14.7)	152	11.8 (10–16.3)	0.6927	0.13	-0.15	**-1.16**
Platelets (10^3^/μL)	289	256 (154–401)	136	249 (153–374)	153	268 (159–439)	**0.0017**	0.25	-0.10	**-1.03**
MPV (fL)	299	8.1 (6.8–9.9)	142	8.1 (6.7–9.7)	157	8.1 (6.8–10.1)	0.2511	0.08	-0.13	**-0.51**
WBC (10^3^/μL)	297	5.4 (3.7–9.2)	141	5 (3.4–8.7)	156	5.8 (3.8–9.9)	**0.0001**	**0.38**	-0.29	**-0.86**
Neutrophils (10^3^/μL)	298	2.43 (1.21–5.18)	141	2.32 (1.12–5.18)	157	2.52 (1.41–5.23)	**0.0026**	0.17	-0.29	-0.05
Monocytes (10^3^/μL)	296	0.18 (0.07–0.42)	142	0.17 (0.07–0.41)	154	0.18 (0.07–0.53)	0.1444	0.11	0.00	**-1.34**
Eosinophils (10^3^/μL)	297	0.23 (0.04–1.72)	142	0.27 (0.04–2.38)	155	0.2 (0.03–1.34)	**0.0077**	0.14	0.02	**2.43**
Basophils(10^3^/μL)	297	0.03 (0.01–0.08)	141	0.03 (0.01–0.08)	156	0.03 (0.01–0.08)	0.8327	0.08	0.00	0.00
Lymphocytes (10^3^/μL)	298	2.3 (1.49–3.72)	141	2.13 (1.44–3.33)	157	2.45 (1.53–4.05)	**< .0001**	**0.40**	-0.16	**-1.27**
CD4 T cell count (cells/μL)	288	832 (491–1381)	137	757 (460–1189)	151	904 (576–1431)	**< .0001**	**0.51**	**-0.51**	**-1.07**

Abbreviations: RBC, red blood cells; WBC, white blood cells; MCH, mean corpuscular hemoglobin; MCHC, mean corpuscular hemoglobin concentration; MCV, mean corpuscular volume; MPV, mean platelet volume; RDW_CV, red cell distribution width as coefficient of variation. Bold Values Indicate Significance: P<0.05; SDR>.3 was used as the critical value for partitioning; BRLL or BRUL>0.375 secondary/alternate criteria for partitioning.

*Range represents 2.5^th^ to 97.5^th^ percentiles.

**P-values indicate comparisons between males and females (Wilcoxon rank sum test).

**Table 2:** Clinical chemistry reference intervals (median and 2.5^th^—97.5^th^ percentiles) derived from healthy adults in Kisumu County, Kenya.

Parameter	Total		Male		Female		*P*-values**	SDR	BRLL	BRUL
	n	Median (range*)	n	Median (range*)	n	Median (range*)				
Alanine aminotransferase (U/L)	292	15.5 (7.9–39.9)	140	17.3 (8.8–45.3)	152	14.2 (7.5–36.8)	**< .0001**	**0.29**	0.16	**1.04**
Creatinine (μmol/L)	298	69 (49–99)	142	80 (59–103)	156	61 (46–76)	**< .0001**	**1.34**	**1.02**	**2.12**

*Range represents 2.5^th^ to 97.5^th^ percentiles.

**P-values indicate comparisons between males and females (Wilcoxon rank sum test). Bold Values Indicate Significance: P<0.05; SDR>.3 was used as the critical value for partitioning; BRLL or BRUL>0.375 secondary/alternate criteria for partitioning.

### Comparison with other regions

Table 3 shows the comparison of the reference values to other Kenyan studies [7, 13, 14, 31] and US derived values [32]. For hematology parameters, there were marked differences when compared to Kericho, Omuse *et al*. findings and the US but more comparable to the Kisumu Incidence Cohort Study (KICoS). The lower limit (LL) of hemoglobin in both men and women was 1.4 and 1.6 times higher than that of Kericho respectively while in women, it was 2.5g/dL lower when compared to US values and Omuse *et al*. (9.5 g/dl vs. 12.0g/dl). About 10% (16/159) of women had hemoglobin values falling below the LL of US and Omuse *et al*. reference values. The MCV LL values for Kericho, Omuse *et al*. and US studies were higher than findings in this study such that 28% (85/299) of the participants had MCV values outside the LL of the US values. However, for females, the MCV LL for this study was more comparable to KICoS which recruited participants from almost a similar population as this study. Platelets were consistently higher in females across three studies and the upper limit (UL) for this study was higher than Omuse *et al*. (154 vs.144) and Kericho (154 vs.120) but comparable to US values (154 vs 150). Eosinophils UL was comparable to Kericho but higher than Omuse *et al*. more so in males (2.34 vs 0.64). Neutrophils in both men and women were higher compared to values from KICoS, Kericho and Omuse *et*.*al*. Similar to Kericho and KICoS, CD4 count was higher in females. For chemistry parameters, the UL of creatinine was 1.3 times lower when compared to the US values but more comparable to KICoS and Kericho such that 3% (8/299) of our participants had values outside our established reference values but were within the US derived reference values. The overall ALT UL was lower than values from Kericho (39.9 U/L vs. 52.0 U/L) but for the females, the UL values were more comparable to values from KICoS.

**Table 3:** Hematology, chemistry, and CD4 T cell count reference intervals (2.5^th^ to 97.5^th^ percentile) in Kisumu County (current study) compared to other regions.

Parameter	Sex	Current	Kericho [14]	KICoS [7]	Kenya^a^ [31]	USA [32]
**Hemoglobin (g/dL)**	M	12.0–17.9	8.3–11.3	12.6–17.2	14.5–18.7	13.5–17.5
	F	9.5–15.3	5.9–10.0	9.0–14.9	12.0–16.5	12.0–16.0
**Hematocrit (%)**	M	38.5–54.0	40–50	38.1–51.6	43.0–55.0	41.0–53.0
	F	32.7–47.3	30–50	28.6–44.2	36.0–49.0	36.0–46.0
**RBC (10^6^/μL)**	M	4.8–6.73	4.4–6.3	4.6–6.6	4.94–6.52	4.5–5.9
	F	4.19–6.29	3.7–5.6	4.0–5.8	4.31–5.76	4.0–5.2
**MCH (pg)**	A	20.7–31.6	22.4–33.5	NA	24.8–32.8	26.0–34.0
**MCV (fL)**	A	59–95	68.8–97.2	NA	75.7–95.6	80.0–100.0
	M	66–95	71.4–98.2	55–98	76.5–95.5	NA
	F	56–98	66.0–95.7	60–94	73.4–95.8	NA
**MCHC (g/dL)**	A	29.9–34.0	32.2–35.3	NA	32.2–35.2	31.0–37.0
**Platelets (10^3^/μL)**	A	154–401	120–411	NA	144–409	150–350
	M	153–374	115–366	126–356	133–356	NA
	F	159–439	124–444	147–454	152–443	NA
**WBC (10^3^/μL)**	A	3.7–9.2	2.8–8.2	NA	3.08–7.83	4.5–11.0
	M	3.4–8.7	2.7–7.5	3.3–9.6	3.13–8.10	NA
	F	3.8–9.9	3.0–9.1	3.7–9.1	2.89–7.72	NA
**Neutrophils (10^3^/μL)**	M	1.12–5.18	0.87–4.32	0.8–3.9	1.02–3.92	NA
	F	1.41–5.23	0.99–5.56	1.3–3.8	1.07–4.42	NA
**Monocytes (10^3^/μL)**	A	0.07–0.42	0.13–0.60	NA	0.14–0.74	NA
	M	0.04–2.38	0.13–5.85	0.2–0.9	0.15–0.76	NA
	F	0.07–0.53	0.16–6.40	0.3–0.8	0.14–0.68	NA
**Eosinophils(10^3^/μL)**	A	0.04–1.72	0.03–1.14	NA	0.04–0.59	NA
	M	0.04–2.38	0.03–1.08	0.1–1.7	0.05–0.64	NA
	F	0.03–1.34	0.03–1.22	0.1–1.3	0.04–0.49	NA
**Basophils (10^3^/μL)**	A	0.01–0.08	0.01–0.08	NA	0.01–0.07	NA
	M	0.01–0.08	0.01–0.09	0.01–0.19	0.01–0.08	NA
	F	0.01–0.08	0.01–0.07	0–0.20	0.01–0.06	NA
**Lymphocytes (10^3^/μL)**	A	1.49–3.72	1.14–3.45	1.4–3.8	1.29–3.40	NA
	M	1.44–3.33	1.12–3.16	1.0–3.5	1.36–3.58	NA
	F	1.53–4.05	1.29–3.96	1.3–3.8	1.22–3.24	NA
**CD4 T cell count(cells/μL)**	A	491–1381	421–1550	NA	NA	404–1612
	M	460–1189	407–1340	462–1306 ^b^	NA	NA
	F	576–1431	483–1651	440–1602 ^b^	NA	NA
**Alanine Aminotransferase (U/L)**	A	7.9–39.9	9.6–52.0	NA	NA	0–35
	M	8.8–45.3	10.8–53.9	8.4–54.7	NA	NA
	F	7.5–36.8	8.6–47.0	7.2–34.1	NA	NA
**Creatinine (μmol/L)**	A	49–99	55–102	NA	NA	0–133
	M	59–103	62–106	69–123	NA	NA
	F	46–76	51–91	57–100	NA	NA

Abbreviations: A, Adult; M, Male; F, Female; KICoS, Kisumu Incidence Cohort Study; RBC, Red Blood Cells; WBC, White Blood Cells, WBC; MCH; mean corpuscular hemoglobin; MCHC, mean corpuscular hemoglobin concentration; MCV, mean corpuscular volume; NA, not available; USA, United States of America.

^a^ Kenyan study by Omuse *et al*. [31]

^b^ CD4 values from the other Western Kenya study by Zeh *et al*. [13].

## Discussion

It is well established that clinical laboratory RIs vary among different populations as they are dependent on multiple factors [6, 8–14, 31–36]. It is therefore essential to develop reference intervals that are region- or population-specific to improve accuracy in interpretation of laboratory results, especially in the context of clinical trials. Misinterpretation of laboratory values has costly consequences such as limiting the use of vaccine or drug, extended periods of research, and mismanagement of patients. In sSA, there is a high burden of HIV [37], tuberculosis [38] malaria [39], emerging infectious diseases [40] and non-communicable diseases [41] which has led to an increase of clinical trials. As such, the development of reference values derived from local SSA populations is needed [1]. This study validates some of the findings by Zeh *et al*. [13] and Collins *et al*. [7] which were performed in rural and urban Western Kenya, respectively. Our study had a mixed population, improving the generalizability of the prior comparable findings. The analyses utilized data of participants in an HIV incidence cohort study that also assessed willingness to participate in future HIV vaccine trials. Although this was a self-selected population, it was representative of participants willing to participate in a clinical trial. The study enrolled adults without HIV from the general population including fisher folk communities and sex workers and it met the recommended CLSI sample size of at least 120 for all clinical parameters assessed. Some of the values observed differed with those derived from regional and US populations.

The established RIs are generally lower than those currently in use which were adopted from the hematology analyzer manufacturer [19]. Hematology parameters showed a similar pattern to other published sSA and US studies [7, 8, 12, 31, 32] where statistically significant gender differences were noted for hemoglobin, hematocrit and RBC with males having higher values than females. This has largely been attributed to menstrual flow in females and the stimulatory effect of testosterone on erythropoiesis [42, 43]. It is worth noting however that while the p-value for statistical difference in gender reference ranges indicates a significant difference in median values between genders, the results do not indicate whether the effect size is of practical significance.

One of the features of acclimatization to high altitude is increased hemoglobin in the blood to allow circulation of oxygen thus higher hemoglobin levels are associated with higher altitude [44, 45]. Interestingly, it was observed that hemoglobin UL and LL values from this study were higher than the ones derived from Kericho [14], despite Kericho being at a higher altitude (2042 m vs. 1131 m above sea level). In this study, 2% (6/299) of participants who would have been classified as anemic based on the established intervals would fall within the hemoglobin RI for Kericho. The variation could be attributed to differences in sample size and other environmental conditions. The study in Kericho was at a single tea plantation whilst in Kisumu, the population cut across fisher folk community, sex workers, and the general population. Low MCV is associated with iron deficiency anemia and in this study the LL for MCV was lower than that derived from a US population [14] and a Kenyan population [31], although there were minimal differences in the median when compared with other Kenyan RI studies [7, 14] and other SSA countries [6, 10–12]. Dietary patterns are diverse in these regions, which could account for the variation; however, this is subject to further review as serum ferritin levels were not assessed in this study and the other Kenyan studies except for Omuse *et al*. [31] where Latent Abnormal Value Exclusion (LAVE) method was applied. The study population in Omuse et al. was largely urban and educated as recruitment was conducted in hospitals, colleges, corporations and shopping malls and thus likely to assess medical care and proper nutrition as opposed to the rural population that was part of this study and KICoS [7], moreover participants with possible sub-clinical disease were excluded from analysis using the LAVE method. Platelets and WBCs were observed to be higher in females compared to males which is synonymous with findings from KICoS [7], Kericho [7], Omuse *et al*. [31], Nigeria [9], Mozambique [8], Uganda [6] and a UK multi-ethnic (Caucasians, Caribbean and Africans) study [46]. Variation in RIs for platelets has been proposed to be due to hormonal differences, and the effect of erythropoietin-release in response to menstruation stimulating megakaryopoiesis [36].In addition, some studies have shown an inverse relationship between hemoglobin level and platelet count [31, 47, 48]. It was also observed that iron supplementation in patients with iron deficient anemia resulted in a drop in platelet count [47]. These observations may explain the gender differences since women have a lower hemoglobin levels compared to men as well as low ferritin [31]. In the UK study [36], the difference in WBC count was attributed to contraceptive use though it is important to note that participants were deemed healthy based on medical history and physical examination and screening for infectious diseases was not performed. In agreement with most published studies, WBC counts in African populations [6, 8, 9, 11, 13, 14] are generally lower compared to those from the US [32]. Eosinophils were higher in males compared to females and the UL for males was also higher than UL values in the other Kenyan studies. This difference could be explained by the fact that the majority of the men in the current study were from the fisher folk community, thus exposure to parasitic infections such as soil-transmitted helminthic infections, malaria, and schistosomiasis [49, 50].

Glomerular filtration rate, a key measurement for kidney function, is calculated using creatinine level, which is also dependent on individual patient factors, time of sample collection and laboratory evaluation methods. Kidney function tests are crucial in determining eligibility and grading adverse events in drug and vaccine trials, thus supporting need for accurate region-specific intervals. The creatinine RI in this study was lower than the other Kenyan studies [13, 14] and US based RIs [32]. If US intervals were used in our population for screening or grading toxicity, three participants with mild elevation (1.1–1.3 times the UL of normal) would be evaluated as normal, thus missing AE reporting and/or enrolling of ineligible participants. For example, in a vaccine or drug trial requiring healthy participants, a participant with mildly elevated creatinine, a signal for early stages of renal failure, could be erroneously enrolled into the study. If the individual worsens upon administration of the investigational product, the elevated creatinine levels could be linked to the vaccine/drug thus limiting its use, pose a safety risk to the participant, and increase research costs in terms of management of AEs.

In agreement with other published data, ALT levels were higher in males [7, 8, 13, 14]. ALT measurement is part of the initial screening for liver damage and hepatitis infection for clinical trial participants and is widely used to monitor liver function in patients on antiretroviral therapy (ART). The ALT UL was comparable to findings from the US [32] but lower than that from Kericho [14], Tanzania [10] and Maputo [8] and higher than findings from Cameroon [51] and Rwanda [34]. If the Kericho RIs were adopted for our population, some participants (5/292), who would have been classified as having mild elevation using our intervals, would be within the established range. Mild elevation of ALT, defined as less than five times of the upper normal limit, is mostly asymptomatic and a common finding in primary care [52]. Other conditions associated with raised ALT levels in asymptomatic patients include fatty liver disease, alcohol abuse, diabetes mellitus, hemochromatosis, α_1_-antitrypsin deficiency, and cirrhosis [52]. These conditions were not assessed in this study.

CD4 count is widely used to assess the extent of immunodeficiency and risk for opportunistic infections and mortality in the management of HIV infection. It has been shown to change with age [53], sex [54, 55], and time of collection [56]. In addition, differences have been noted between different populations in Europe [57], Asia [58], and Africa [6, 34, 59, 60]. In this study, absolute count CD4 T cell counts were higher in females compared to males which is similar to findings from studies in other African and Asian countries [6, 8, 13, 14, 34], which has been associated with hormonal differences [61]. The median CD4 count in this cohort is comparable to those reported from the Central African Republic [12] and Malawi [56] but is higher than median values from Tanzania [10], Nigeria [62], Uganda [6], and Ethiopia [11]. These variations in CD4 count could be attributed to environmental factors, genetics, ethnic differences, analyzers used, and/or study design [55, 60, 62]. In light of the ‘test and treat’ strategy in Kenya, initiation of ART is no longer based on CD4 count but it is important to know the RIs as this may influence prognosis and treatment response and is used to determine the timing of co-infection prophylaxis initiation.

A number of limitations were noted in this study. First, as per CLSI guidelines for establishing clinical RIs, factors influencing laboratory indicators such as environmental, genetic, and social habits such as dietary patterns, alcohol abuse, and smoking should be considered but these factors were not evaluated for study participants. Secondly, even though thorough medical examination including laboratory evaluation for some infections (e.g. malaria, hepatitis, and syphilis) was done, not all conditions known to affect hematology and chemistry parameters were assessed or excluded (e.g. alcohol and substance use, lactation and genetic disorders) as well as analytes such as ferritin that would help identify participants with latent anemia and other sub-clinical diseases. Additionally, due to limited resources, not all chemistry parameters (e.g. bilirubin, cholesterol, glucose, albumin, liver-associated enzymes) were assessed. These are useful in the screening and diagnosis of diabetes and liver disorders and assessing hepatotoxicity of drugs (ART and anti-tuberculous drugs) leading to potentially missed conditions that could influence our observed results and confound our conclusions.

In conclusion, this study supports the findings from other published studies that show significant differences between RIs derived from the US and African populations. Importantly, it highlights the differences in key parameters such as hemoglobin and ALT between our findings and other Kenyan studies, so that if our site adopted the other Kenyan RIs, a number of participants could be excluded or erroneously enrolled, further supporting the importance of locally-defined RIs. In addition, whilst participants or patients could undergo extensive unnecessary investigations that create anxiety and are also costly in an already resource-constrained setting, other patients may miss undergoing further evaluation and deteriorate later. With the rise in non-communicable diseases in Africa [1, 2] such as diabetes, hypertension, renal disease, and cancer, screening for early detection and treatment has been the mainstay for prevention. Therefore, accurate clinical laboratory RIs will likely enhance effectiveness of screening programs. Additionally, with the increase in clinical trials in sSA [2, 3, 63], accurate RIs will be beneficial in improving patient care, scientific validity and reduction of research costs.
